# Electrochemically Grown Ultrathin Platinum Nanosheet Electrodes with Ultralow Loadings for Energy-Saving and Industrial-Level Hydrogen Evolution

**DOI:** 10.1007/s40820-023-01117-2

**Published:** 2023-06-03

**Authors:** Lei Ding, Zhiqiang Xie, Shule Yu, Weitian Wang, Alexander Y. Terekhov, Brian K. Canfield, Christopher B. Capuano, Alex Keane, Kathy Ayers, David A. Cullen, Feng-Yuan Zhang

**Affiliations:** 1 Nanodynamics and High-Efficiency Lab for Propulsion and Power, Department of Mechanical, Aerospace & Biomedical Engineering, UT Space Institute (University of Tennessee-Knoxville), Tullahoma, TN 37388 USA; 2 Center for Laser Applications, UT Space Institute (University of Tennessee-Knoxville), Tullahoma, TN 37388 USA; 3 Nel Hydrogen, Wallingford, CT 06492 USA; 4 Oak Ridge National Laboratory, Center for Nanophase Materials Sciences, Oak Ridge, TN 37831 USA

**Keywords:** Seeding layer, Electrochemically grown Pt nanosheet, Ultralow loadings, High catalyst utilization, Hydrogen evolution

## Abstract

Nanostructured catalyst-integrated electrodes with remarkably reduced catalyst loadings, high catalyst utilization and facile fabrication are urgently needed to enable cost-effective, green hydrogen production via proton exchange membrane electrolyzer cells (PEMECs). Herein, benefitting from a thin seeding layer, bottom-up grown ultrathin Pt nanosheets (Pt-NSs) were first deposited on thin Ti substrates for PEMECs via a fast, template- and surfactant-free electrochemical growth process at room temperature, showing highly uniform Pt surface coverage with ultralow loadings and vertically well-aligned nanosheet morphologies. Combined with an anode-only Nafion 117 catalyst-coated membrane (CCM), the Pt-NS electrode with an ultralow loading of 0.015 mg_Pt_ cm^−2^ demonstrates superior cell performance to the commercial CCM (3.0 mg_Pt_ cm^−2^), achieving 99.5% catalyst savings and more than 237-fold higher catalyst utilization. The remarkable performance with high catalyst utilization is mainly due to the vertically well-aligned ultrathin nanosheets with good surface coverage exposing abundant active sites for the electrochemical reaction. Overall, this study not only paves a new way for optimizing the catalyst uniformity and surface coverage with ultralow loadings but also provides new insights into nanostructured electrode design and facile fabrication for highly efficient and low-cost PEMECs and other energy storage/conversion devices.

**Supplementary Information:**

The online version contains supplementary material available at 10.1007/s40820-023-01117-2.

## Highlights

- 4-nm-thick Pt nanosheets (Pt-NSs) are electrochemically grown on Ti substrates for hydrogen evolution reactions.
- Highly uniform Pt-NS surface coverage with an ultralow loading of 0.015 mg_Pt_ cm^−2^ is achieved.
- 99.5% catalyst savings and about 237-fold higher catalyst utilization are demonstrated.

**Supplementary Information:**

The online version contains supplementary material available at 10.1007/s40820-023-01117-2.

## Graphical abstract

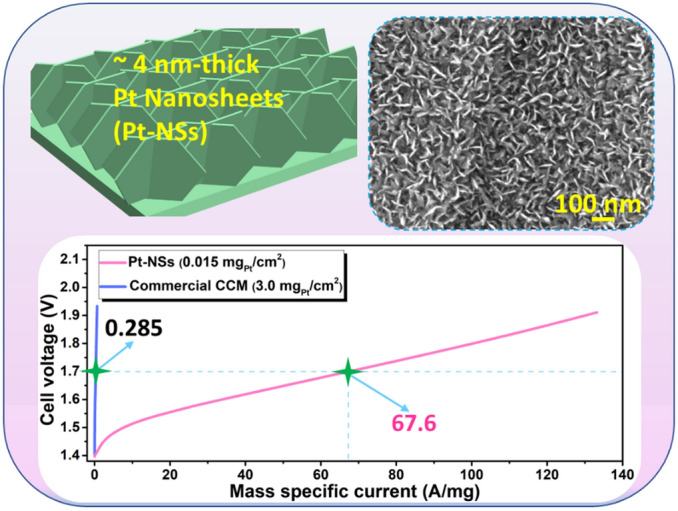

## Introduction

Currently, most hydrogen is produced from fossil fuels, leading to more than 2% of carbon dioxide emissions in the world [1–7]. An efficient method to reduce the carbon dioxide emission from hydrogen production is using water electrolysis with renewable energy as the electricity source. Due to their high energy efficiency, quick response, compact design, and gas crossover reduction, proton exchange membrane electrolyzer cells (PEMECs) are considered the most promising technology for green hydrogen production [8–13]. Nevertheless, scarce and expensive platinum-based materials are typically used as catalysts for hydrogen evolution reactions (HERs) occurring at the cathode in PEMECs. Normally, high Pt loadings of 1 ~ 3 mg_Pt_ cm^−2^ are required for the conventional catalyst-coated membrane (CCM) to ensure stable cell performance [14–18], resulting in thick catalyst layers of more than 10 µm. However, Mo et al*.* [19] demonstrated that most of the catalysts in the conventional CCM are underutilized due to the involved ionomer with limited electron conductivity. In addition, the conventional CCM design normally involves multiple-step fabrication processes and elaborate equipment, which is time-consuming and high cost. Therefore, there is an urgent need to develop high-performance electrodes with reduced Pt loadings, improved catalyst utilization and an efficient fabrication method.

To reduce the Pt loading, one efficient strategy is to directly deposit catalysts on the gas diffusion layer (GDL) to form gas diffusion electrodes (GDEs). So far, various Pt-based catalysts have been grown on the GDL and utilized as HER electrodes for the PEMEC. For example, Kang et al*.* [20] sputtered Pt films with low Pt loadings (0.032 ~ 0.193 mg_Pt_ cm^−2^) on thin and tunable liquid/gas diffusion layers (TTLGDLs) as the cathode electrodes, achieving 58 times higher mass activity than the commercial CCM with a Pt loading of 3 mg_Pt_ cm^−2^ at 1.6 V. Laube et al*.* [21] used an atomic layer deposition method to deposit catalysts on the GDLs to reduce the catalyst loadings, achieving a Pt loading of 0.28 mg_Pt_ cm^−2^ on the cathode. However, lower Pt loadings with fine nanostructures are desired to further decrease the catalyst cost with comparable or even improved performance since more active sites would be exposed with fine nanostructured catalyst layers. Moreover, compared to the flat or film-like catalyst layer, the nanostructured catalyst layer could not only offer larger surface areas, exposing more active sites but also achieve favorable bubble detachment due to the weaker bubble adhesive force [22]. Currently, some nanostructured Pt catalysts have been prepared for the PEMEC. For example, Li et al*.* [23] prepared electrodes with different Pt structures for the HER and revealed that a pine-shaped Pt nanostructured electrode with a loading of 0.215 mg_Pt_ cm^−2^ showed superior performance to both a Pt nanosphere-based electrode and a flat Pt coating-based electrode. Meanwhile, smaller bubble detachment sizes are observed for the pine-shaped Pt nanostructured electrode. Xie et al*.* [24] developed fine Pt nanowires with an average diameter of about 5 nm via a wet-chemical method, achieving favorable bubble detachment and excellent performance in the PEMEC. A low Pt loading of 0.1 mg_Pt_ cm^−2^, a low cell voltage of 1.643 V and a higher efficiency of 90.08% at 1 A cm^−2^ were verified when combined with a Nafion 115 membrane, which are superior to those of the conventional CCM (1.846 V and 80.17%). Meanwhile, about 15 times catalyst savings are achieved with the fabricated Pt nanowire electrode. Additionally, Park et al*.* [25] fabricated flower-like Pt and polyhedral Pt particles with low loadings (0.020 ~ 0.071 mg_Pt_ cm^−2^) on carbon papers via pulse electrodeposition as cathode electrodes for the PEMEC. However, due to the poor catalyst surface coverage, high cell voltages were observed even with a thin (50 µm) Nafion 212 membrane. Overall, to accelerate the large-scale application of the PEMEC, highly efficient cathode electrodes with further decreased catalyst loadings, fine nanostructured catalyst layers, improved surface coverage under ultralow loadings, and efficient and easy scale-up fabrication are desired.

In this work, bottom-up grown Pt nanosheets (Pt-NSs) with ultralow loadings and good surface coverage were first deposited on thin tunable liquid/gas diffusion layers (TTLGDLs) (thickness: 25 µm, pore size: 100 µm, porosity: 40%) via a surfactant- and template-free and fast electrochemical growth process at room temperature, exhibiting ultrathin thickness of only about 4 nm and vertically well-aligned nanosheet morphologies. Compared to conventional Pt nanoparticle-based catalysts and CCM design, the catalyst and electrode fabrication processes would be greatly simplified. When combined with an anode-only Nafion 117 CCM, the Pt-NS integrated ultrathin electrode with an ultralow loading of 0.015 mg_Pt_ cm^−2^ could achieve superior cell performance compared to the commercial CCM, saving 99.5% Pt catalyst and increasing catalyst utilization of more than 237-fold. Moreover, the highly efficient, facile, and cost-effective electrochemical growth process exhibits great potential for industrial application. Additionally, compared to conventional thick GDEs (several hundred μm), the ultrathin electrode shows a dramatically decreased thickness of about only 25 μm, which is material/weight/volume/cost-saving and would benefit a compact stack design in future. Overall, the seed-assisted Pt-NS integrated electrode could not only guide the preparation of a uniform catalyst surface coating but also accelerate the PEMEC industrial application. Meanwhile, it could be easily extended to fuel cells and other electrochemical energy storage/conversion systems.

## Experimental Section

### Platinum Nanosheet Integrated Electrode Fabrication

With a rapid and facile electrochemical growth process at room temperature, template-free and surfactant-free Pt nanosheets with ultralow loadings and fine surface coverage were deposited on titanium-based TTLGDLs. Before the electrochemical growth process, TTLGDLs were treated in oxalic acid to remove the surface oxide layer. Afterward, with a sputtering speed of ~ 0.34 nm s^−1^ and a sputtering time of 5 s, about a 2-nm-thick Pt nanoparticle layer was sputtered on TTLGDLs, serving as a seeding layer. The electrochemical growth process was performed in a three-electrode system, in which a Pt foil, a saturated calomel electrode (SCE) and the TTLGDL were used as the counter electrode, reference electrode and working electrode, respectively. With an electrolyte containing 5 mmol L^−1^ H_2_PtCl_6_ (Alfa Aesar) and 0.5 mol L^−1^ HCl (Alfa Aesar), fine Pt-NSs with ultralow loadings of 0.025 and 0.015 mg_Pt_ cm^−2^ were quickly (< 25 s) and uniformly deposited on TTLGDLs at –1.0 V versus SCE at room temperature. Meanwhile, higher Pt-NS loadings of 0.05 and 0.14 mg_Pt_ cm^−2^ were also prepared to investigate the Pt-NS size and thickness variation along with the loading increase. Additionally, without the seeding layer, several samples with different Pt-NS loadings (0.025, 0.07 and 0.14 mg_Pt_ cm^−2^) were also prepared for comparison. Moreover, sputtered Pt nanoparticles (PtNP) and commercial Pt black (Fuel Cell Store) were also prepared on the Ti substrate for *ex-situ* hydrogen evolution reaction (HER) performance comparison.

### Material Characterization

A field emission JSM-IT700HR scanning electron microscope (SEM) with energy-dispersive X-ray spectroscopy (EDS) was used to characterize the morphology and composition of the samples. A Rigaku SmartLab X-ray diffraction (XRD) system was used to investigate the crystalline structure of the sample. The crystalline structure of Pt-NS catalysts was investigated by scanning transmission electron microscopy (STEM) on a probe-corrected JEOL NEOARM operated at an accelerating voltage of 80 kV.

### Ex situ Electrochemical Measurements

A typical three-electrode system with a saturated calomel electrode (SCE) as the reference electrode and a Pt foil as the counter electrode was used to evaluate the ex situ HER performance of the prepared electrodes. All tests were performed on a potentiostat (SP300, Bio-Logic) in 0.5 M H_2_SO_4_ at room temperature. Based on the Nernst equation: E_RHE_ = E_SCE_ + 0.26 V, all the recorded potentials, E_SCE_, were converted to a reversible hydrogen electrode (RHE). Before the tests, the electrolyte was saturated with Ar for 0.5 h to remove the oxygen. The linear sweep voltammetry (LSV) curves were recorded from 0 to − 0.5 V versus RHE with a scan rate of 5 mV s^−1^. The electrochemical impedance spectroscopy (EIS) plots were recorded within a frequency range of 400 kHz ~ 50 mHz. The “ZFit” impedance fitting tool within the EC-Lab software was utilized to analyze the EIS results with the Randomize + Simplex method. An equivalent circuit model with a resistance in series (*R*_*Ω*_) and two parallel *Q/R* components in high-frequency and low-frequency regions was chosen to fit the Nyquist plots. Based on the fitting, different parameters can be directly derived. The high-frequency resistance (HFR) plots during the PEMEC test were recorded under the high frequency of ~ 6 kHz by using the Staircase Galvano Electrochemical Impedance Spectroscopy (SGEIS) scan with the EC-Lab software. The current density range used for the scans was between 0 and 2 A cm^−2^. Meanwhile, the visualization system setup consisting of a camera, lens and lighting can be found in our previous publications [26–30].

### Cell Performance Evaluation of the PEMEC

All cell tests were carried out in a PEM electrolyzer cell with an active area of 5 cm^2^ and a working temperature of 80 °C. The water flow rate at the anode side is 20 mL/min, and the pressure at both the anode and cathode is 1 atm. The prepared Pt-NS electrode was used as the cathode and an anode-only Nafion 117 membrane (175 µm thick, Nel Hydrogen) with 2.0 mg_Ir_ cm^−2^ IrO_x_ was used as the anode. A commercial CCM with 3.0 mg_Ir_ cm^−2^ IrO_x_ as the anode and 3.0 mg_Pt_ cm^−2^ Pt black as the cathode was used as a baseline. All the test plots were recorded on a potentiostat (VSP/VMP3B-100, Bio-Logic).

## Results and Discussion

### Morphology and Composition of the Pt-NS Electrode

**Fig. 1:**
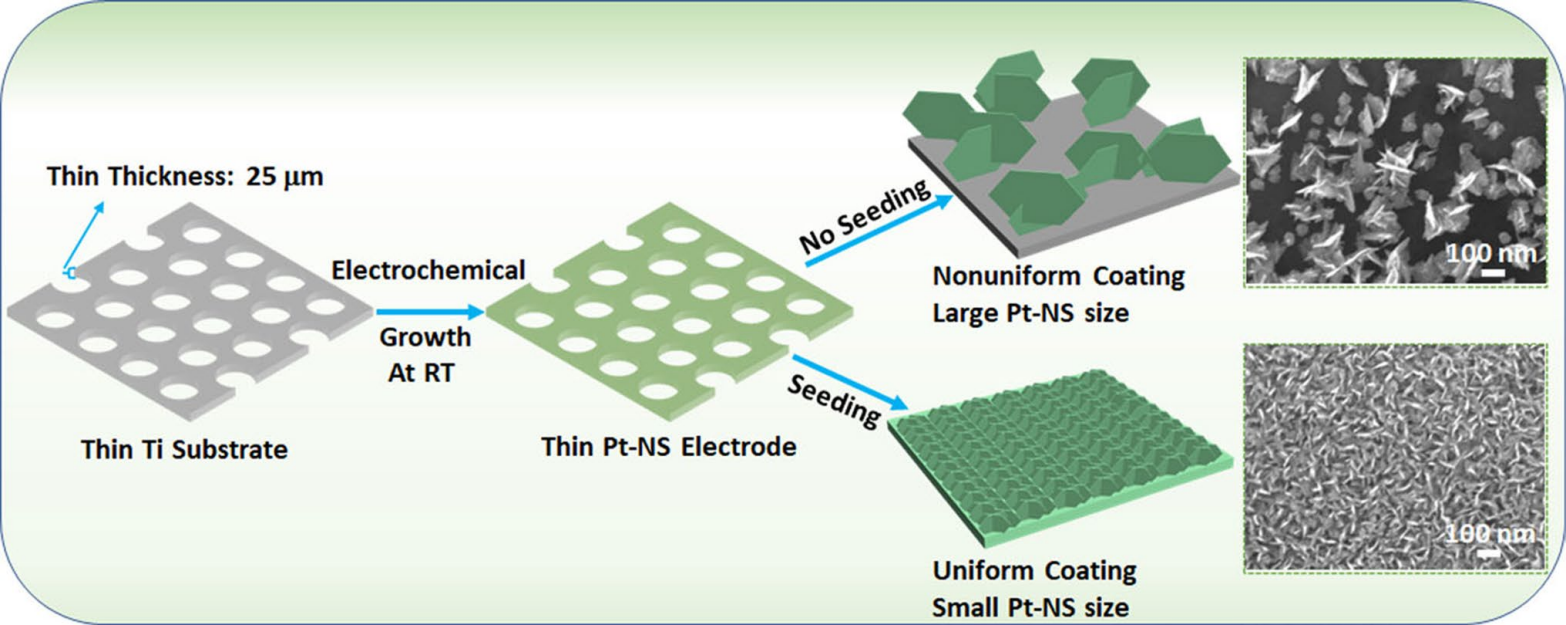
Schematic of the Pt-NS fabrication on the thin Ti substrate (with seeding and no seeding, respectively) via a highly efficient and facile electrochemical growth process at room temperature

**Fig. 2:**
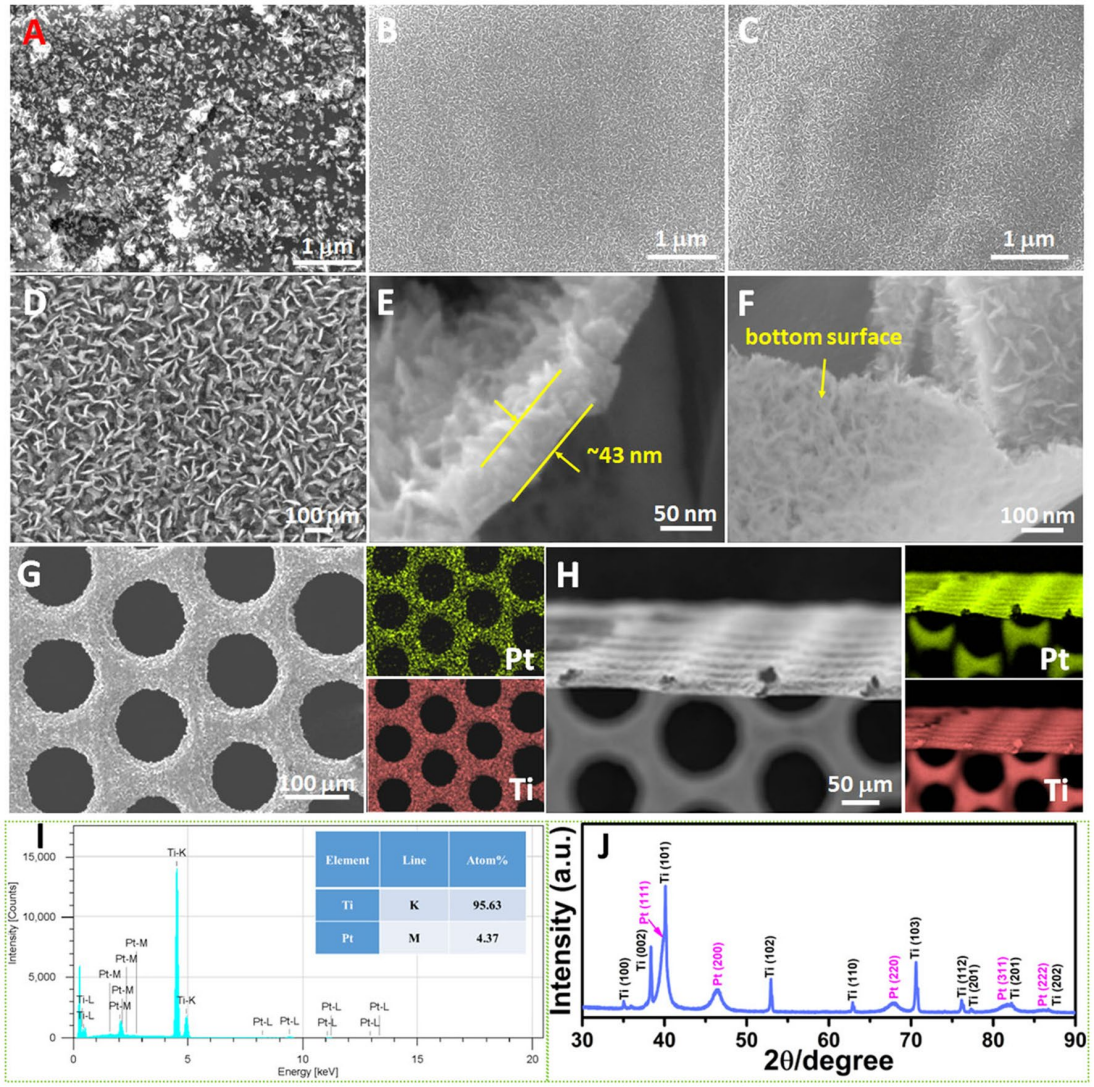
**A**–**C** Low magnification SEM images of Pt-NSs with different loadings: **A** 0.025 mg_Pt_ cm^−2^ (without the seeding layer), **B** 0.025 mg_Pt_ cm^−2^ (with the seeding layer), **C** 0.015 mg_Pt_ cm^−2^ (with the seeding layer); **D**–**F** High magnification SEM images of Pt-NSs based on 0.025 mg_Pt_ cm^−2^; **G** Top-view and **H** cross section view SEM–EDS mapping images of the Pt-NS electrode based on 0.025 mg_Pt_ cm^−2^; The related **I** EDS spectra; **J** XRD pattern of the Pt-NSs

As illustrated in Fig. 1**,** Pt-NSs can be easily grown on the thin Ti substrate (~ 25 µm) via a highly efficient, cost-effective, template-free, and surfactant-free electrochemical growth process at room temperature. With or without a thin Pt seeding layer, different nanosheet structures with different sizes and coverages are observed on the thin substrate. Specifically, in the absence of the seeding layer, a nonuniform Pt-NS coating with nanoparticles and large nanosheet sizes is observed on the substrate surface, while with the thin Pt seeding layer, a highly uniform Pt-NS coating with small nanosheet sizes is formed. As shown in Fig. 2A, without the Pt seeding layer, large Pt nanosheet assemblies along with some small Pt nanoparticles are nonuniformly deposited on the substrate surface at a Pt-NS loading of 0.025 mg_Pt_ cm^−2^. When increasing the catalyst loading to 0.07 mg_Pt_ cm^−2^, Pt nanosheet assemblies grow larger (Fig. S1). Meanwhile, larger nanosheets with sizes of 100 ~ 170 nm and an average thickness of about 12 nm are observed. When further increasing the loading to 0.14 mg_Pt_ cm^−2^, the Pt-NSs still do not fully cover the Ti substrate. As shown in Fig. S2, some nanoparticles and bare Ti surfaces can be still seen. However, benefiting from the thin seeding layer of Pt nanoparticles with an average size of about 5 nm (Fig. S3A, B), vertically aligned Pt-NSs with uniform surface coverage are observed with an ultralow loading of 0.025 mg_Pt_ cm^−2^ (Fig. 2B). Moreover, the resultant fine Pt-NSs show much smaller sizes of 28 ~ 55 nm and thinner nanosheets of about 4 nm compared to those without the thin seeding layer. By merely shortening the electrochemical growth time, a further decreased Pt-NS loading of only 0.015 mg_Pt_ cm^−2^ was also prepared. As shown in Fig. 2C, at the loading of 0.015 mg_Pt_ cm^−2^, fine and vertically aligned nanosheets with uniform surface coatings are still formed on the substrate, showing a smaller thickness of about 3 nm and similar sizes to the 0.025 mg_Pt_ cm^−2^-based nanosheets. Hence, a thin seeding layer is a prerequisite for highly uniform Pt-NS coatings on the Ti substrate with ultralow loadings. In addition, with the help of the seeding layer, the nanosheet size and thickness can be well-modulated by merely tuning the electrochemical growth time. As shown in Fig. S3C, D, when the Pt-NS loading increases to 0.05 and 0.14 mg_Pt_ cm^−2^, the Pt-NS sizes increase to 50 ~ 100 and 100 ~ 120 nm, showing thicknesses of about 10 and 15 nm, respectively. Notably, along with the loading increase, the Pt nanosheets maintain the fine, vertically aligned nanosheet morphologies, growing into larger sizes and providing a large surface area.

SEM images of the Pt-NS catalyst layer (0.025 mg_Pt_ cm^−2^) from the top view (Fig. 2D) and tilted view (Fig. 2E) further reveal the fine and vertically well-aligned Pt nanosheets. In addition, as demonstrated in Fig. 2E, a relatively uniform thickness of about 43 nm for the Pt-NS catalyst layer is presented, successfully achieving a nanoscale catalyst layer with fine nanosheet structures. Notably, the exposed bottom surface of the Pt-NS catalyst layer shows nanosheet arrays with some porous structures, demonstrating a bottom-up Pt nanosheet growth process (Fig. 2F). The bottom-up Pt-NS growth should be due to the thin seeding layer, which could provide homogeneous nucleation sites and assist the Pt nanosheet to grow from the bottom. The seed-assist bottom-up Pt-NS growth allows high surface uniformity of the fine Pt-NSs with ultralow loadings showing nanoscale catalyst layer, small nanosheet sizes, and thin nanosheet thicknesses, which could offer large surface areas and benefit the electrochemical reaction. These results differ from other publications [23, 25, 31–34], in which nonuniform Pt coatings are observed on substrate surfaces. For example, bare areas are observed for the pine-shaped Pt nanostructured electrode even with a Pt loading up to 0.215 mg_Pt_ cm^−2^ [23]. In addition, low Pt surface coverage is also observed for the porous Pt nanoflowers with a loading of 0.200 mg_Pt_ cm^−2^ prepared by a template-free electrodeposition process [33]. Liu et al. [34] electrodeposited different Pt nanostructures (spherical, nanocube, or willow-like) on glassy carbon substrates by tuning the deposition potentials of a square-wave potential electrodeposition process. However, low surface coverages were observed for all those nanostructured Pt catalysts. Hence, the seed-assisted Pt-NS electrochemical growth with high surface coverage, fine nanosheet morphology, and ultralow loadings shows great advantages to develop highly efficient catalysts at low cost.

The top view and cross section view SEM–EDS mapping images (Fig. 2G, H) confirm the full surface coverage of the Pt-NS catalyst layer on the TTLGDL substrate and only about 25 μm thickness for the whole electrode. Unlike conventional thick electrodes with several hundred-μm thicknesses, the Pt-NS integrated ultrathin electrode with ultralow catalyst loadings would be beneficial for a more compact and low-cost cell and stack design in future. As shown in the EDS results (Fig. 2I), about 4.37% Pt catalysts are deposited on the Ti substrate surface with an ultralow loading of 0.025 mg_Pt_ cm^−2^. Meanwhile, as seen from the XRD pattern (Fig. 2J), apart from the peaks of the Ti substrate, five 2θ peaks assigned to Pt are observed at about 39.8°, 46.4°, 67.9°, 81.5° and 86.2°, which are ascribed to Pt(111), Pt(200), Pt(220), Pt(311), and Pt(222), respectively [35–37]. These results match well with the face-centered cubic (fcc) structure of crystalline Pt.

### Growth Mechanisms of the Pt-NSs

**Fig. 3:**
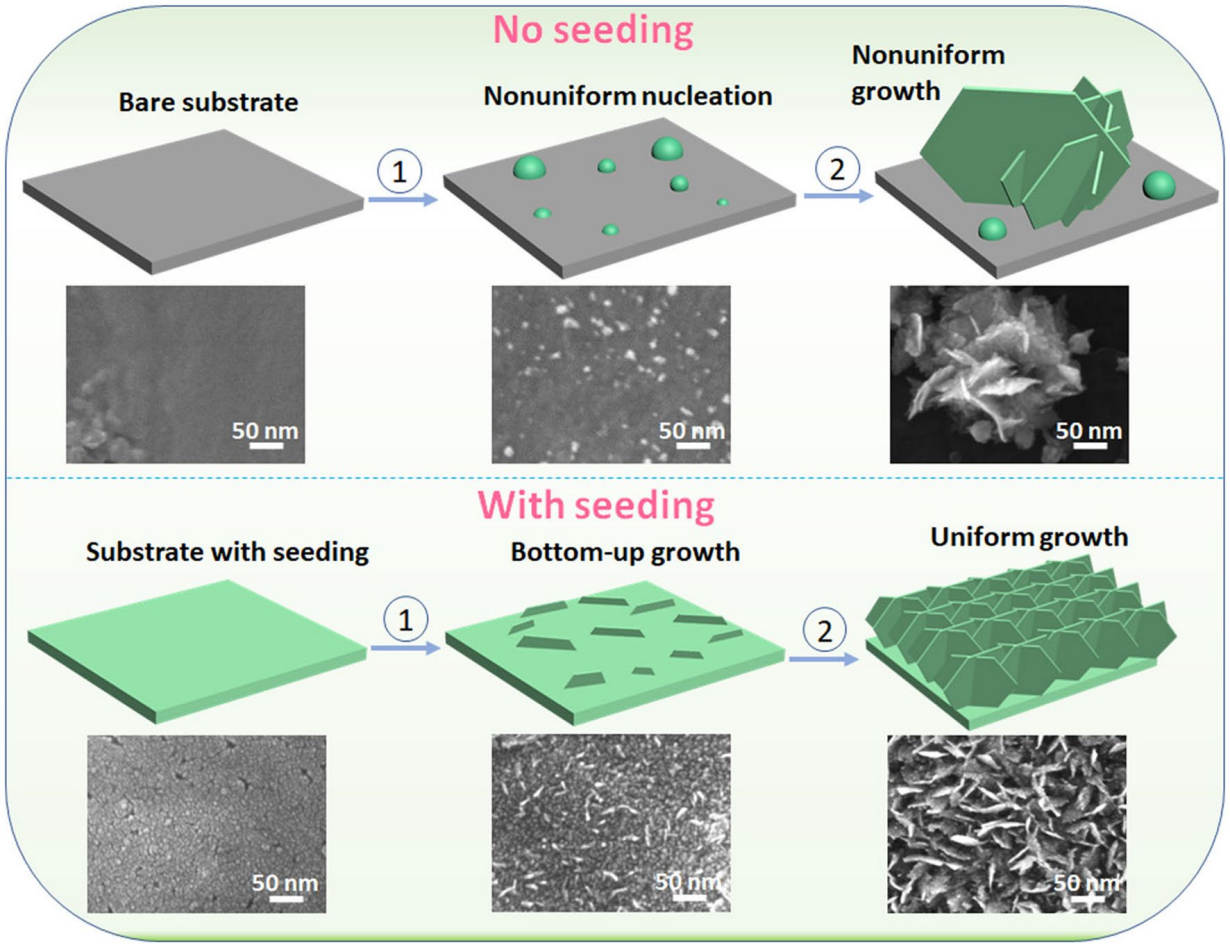
Pt-NS growth mechanisms without (top part) and with (bottom part) the seeding layer

To study the growth mechanism of Pt-NS on the Ti substrate, we prepared two types of Pt-NS samples with and without the seeding layer. For the seeding layer case, by adjusting the electrodeposition times, Pt-NS samples with different loadings of 0.015, 0.025, 0.05 and 0.15 mg_Pt_ cm^−2^ were prepared. For the case of without the seeding layer, three samples with different Pt-NS loadings (0.025, 0.07 and 0.14 mg_Pt_ cm^−2^) were also prepared for comparison. With an increase in electrodeposition time, there is a concurrent increase in the loading of Pt-NS, which is accompanied by a noticeable change in morphology. Based on SEM characterizations of each type of sample at different loadings (corresponding to different electrodeposition times), we proposed the possible Pt-NS growth mechanism. Two processes are involved during the electrodeposition procedure. One is a diffusion process, in which Pt ions are transported from the bulk solution through the diffusive layer toward the electrode; the other is an activation process, in which the Pt ions are reduced on the electrode surface [38]. The possible mechanisms of the Pt nanosheet formation with or without the Pt seeding layer are shown in Fig. 3 and discussed as follows. As reported in the previous studies [39, 40], the nanosheet formation could be attributed to the applied large electrodeposition overpotential. Under a large electrochemical growth overpotential of –1.0 V versus SCE, the Pt species in the electrolyte are quickly reduced into Pt crystal nuclei in the initial stage. During this stage, Pt species are consumed because of the fast reduction process, while the Pt ion diffusion to the electrode could compensate for the consumption. However, due to the high reduction rate under the large overpotential, the consumed Pt ions cannot be quickly compensated, leading to a diffusion-controlled process. In this case, the edges of Pt crystal nuclei preferentially grow faster than the other parts, resulting in nanosheet morphology formation for the subsequent growth stage. However, the Pt crystal nuclei are distributed nonuniformly on the non-seeded Ti substrate, probably due to different energy barriers for nucleation in different locations across the whole substrate surface and differences in the local surface roughness. Favorable nucleation usually initiates at the rougher surface with higher binding energy sites for Pt species. The nonuniform nucleation on a non-seeded substrate may lead to different nanosheet growth rates in different locations. Hence, without a thin Pt seeding layer, large Pt-NSs with nonuniform surface coverage and thick nanosheets are formed on the substrate. Meanwhile, some small nanoparticles are also observed on the substrate. However, a thin Pt seeding layer could provide more uniform and denser nucleation sites, which can effectively regulate the nucleation rate to successfully achieve bottom-up growth. As a result, with the seeding layer, highly uniform nanosheet growth with ultrathin nanosheet thickness, small nanosheet sizes, and fine vertically aligned nanosheet morphologies are obtained across the entire substrate surface, unlike a seed-free electrochemical growth process. Our research work is still ongoing to better understand the Pt-NS growth mechanism on titanium substrate by employing potential *in situ* characterization techniques to monitor the real-time Pt-NS growth process.

**Fig. 4:**
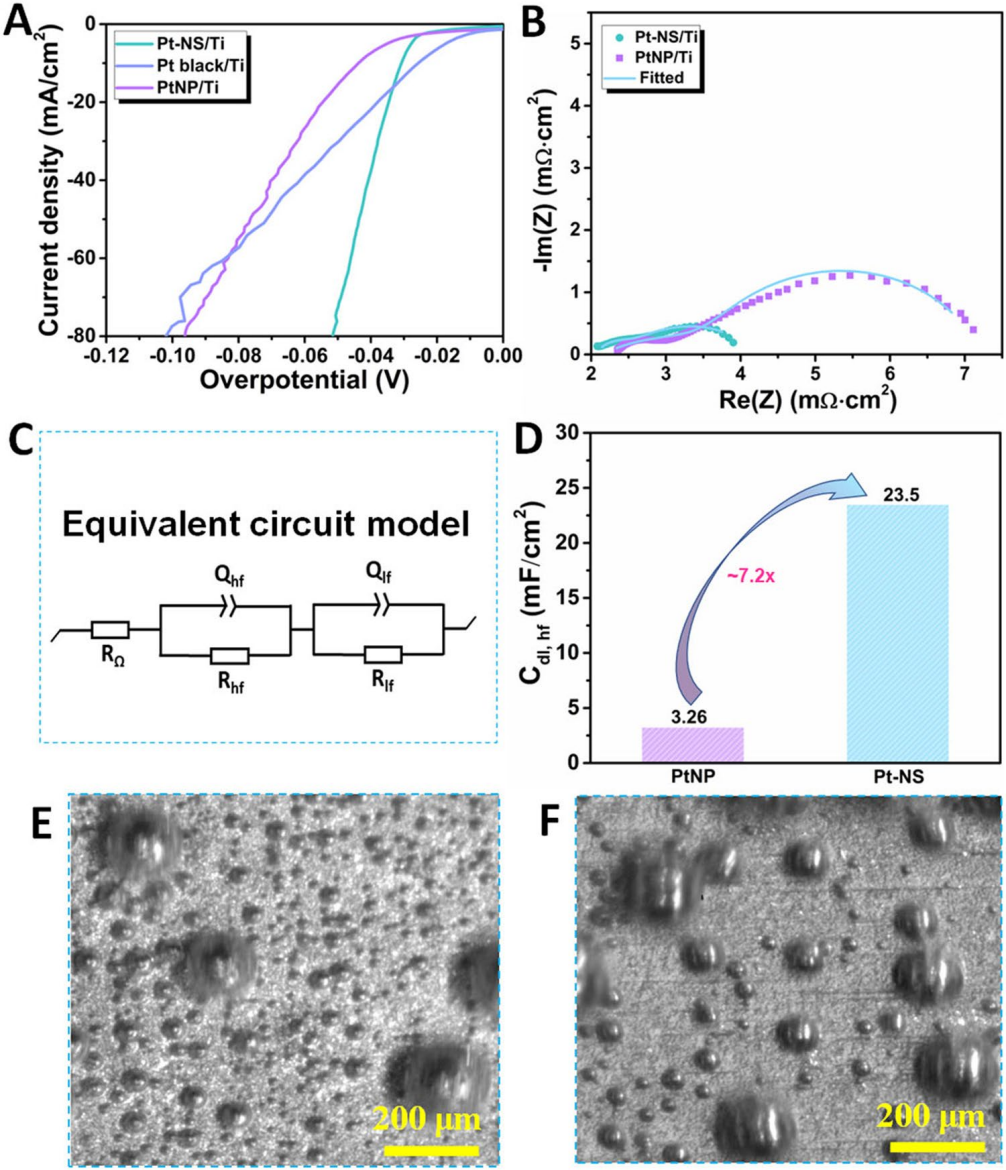
*Ex situ* electrochemical characterizations of the Pt-NS/Ti electrode via a typical three-electrode system in 0.5 M H_2_SO_4_. at room temperature. **A** IR-corrected HER polarization curves with a scan rate of 5.0 mV s^−1^; **B** EIS plots recorded at − 40 mV; **C** The fitting equivalent circuit model; **D** Double-layer capacitance comparison; High-speed visualization of hydrogen bubble dynamics on **E** the Pt-NS electrode and **F** the PtNP electrode, respectively, at 200 mA cm.^−2^ (Movies S1 and S2)

To evaluate the HER performance of the Pt-NS, electrochemical measurements in 0.5 M H_2_SO_4_ in a typical three-electrode system were performed at room temperature. For comparison, sputtered Pt nanoparticles (PtNP) and commercial Pt black with the same Pt loading were also tested under the same conditions. All potentials from the three-electrode system are iR-corrected and normalized to RHE. As shown in Fig. 4A, the Pt-NS electrode shows a low overpotential of − 30 mV at − 10 mA cm^−2^, close to that of the commercial Pt black (− 26 mV) and lower than that of the PtNP (− 44 mV). Notably, larger than about − 16 mA cm^−2^, the Pt-NS shows superior HER performance to the commercial Pt black. At a current density of − 80 mA cm^−2^, the Pt-NS shows a low overpotential of only − 52 mV (Pt black: − 102 mV), implying that the Pt-NS with fine nanosheet morphologies could offer rich active sites to decrease the overpotential within the high current density range. To investigate the hydrogen adsorption kinetics of ionomer-free Pt catalysts with different morphologies, the EIS plots of Pt-NS and PtNP were recorded at − 40 mV.

As revealed from the Nyquist plots (Fig. 4B), two arcs in the high-frequency (HF) and low-frequency (LF) ranges are observed, indicating a two-time-constant process [29, 41, 42]. To fit Nyquist plots, a model with a resistance in series (*R*_*Ω*_) and two parallel connections of resistance and constant phase elements assigned to the HF (*R*_*hf*_ and *Q*_*hf*_) and LF (*R*_*lf*_ and *Q*_*lf*_) ranges, respectively, was used, as shown in Fig. 4C. The fitted data are presented in Table S1, in which similar ohmic resistances are observed for both Pt-NS and PtNP, and this is reasonable since the two electrodes were both tested in 0.5 M H_2_SO_4_ at room temperature. The resistance summaries of R_*hf*_ and *R*_*lf*_ of the Pt-NS are lower than those of the PtNP, indicating a faster HER faradaic process. Meanwhile, the double-layer capacitances for HF (*C*_*dl, hf*_) and LF (*C*_*dl, lf*_) of the two electrodes were also derived from the plot fitting, which can be used to represent the active site number of the catalysts [43–46]. As mentioned above, the Nyquist plots show a two-time-constant process, in which the *C*_*dl, hf*_ at HF with a smaller time constant belongs to a “fast process,” while the *C*_*dl, lf*_ at LF is a “slow process,” since a larger time constant is needed for the reaction sites [29, 47, 48]. Hence, a higher *C*_*dl, hf*_ value indicates more active sites are exposed for the “fast process” and a lower *C*_*dl, lf*_ value implies that fewer active sites are assigned to the “slow process.” As shown in Fig. 4D, the Pt-NS exhibits a higher *C*_*dl, hf*_ value of 23.5 mF cm^−2^, which is about 7.2-fold higher than that of the PtNP (3.26 mF cm^−2^), indicating that abundant electrochemical reaction active sites are exposed for the Pt-NS electrode. Meanwhile, compared to the PtNP, a lower *C*_*dl, lf*_ value is obtained for the Pt-NS. Overall, the Pt-NS with ultrathin, vertically well-aligned nanosheets yields abundant active sites for HERs, resulting in an excellent performance.

To further validate the rich active sites of the Pt-NS electrode, *in situ* visualization was performed with a high-speed and microscale visualization system to investigate the bubble dynamics at a current density of 200 mA cm^−2^ (Movie S1). For comparison, the bubble dynamics on the PtNP electrode were also captured at the same current density (Movie S2). It is clearly shown that compared to the PtNP electrode, much more bubble detachment sites are observed on the Pt-NS electrode (Fig. 4E, F). Notably, the more numerous bubble detachment sites on the Pt-NS electrode mean that enhanced total reaction zones are achieved on the electrode surface, reflecting the abundant reaction sites to some extent. In addition, much smaller bubbles are observed on the Pt-NS electrode surface, indicating weaker bubble adhesive force on the electrode, which could achieve favorable bubble detachment. As reported in previous references [23, 24], compared to the catalyst layer with flat surfaces, the nanostructured catalyst layer could decrease the bubble contact area on the electrode surface, successfully achieving weaker bubble adhesive force and quicker bubble removal with smaller sizes.

### Pt-NS Characterization in PEMECs

**Fig. 5:**
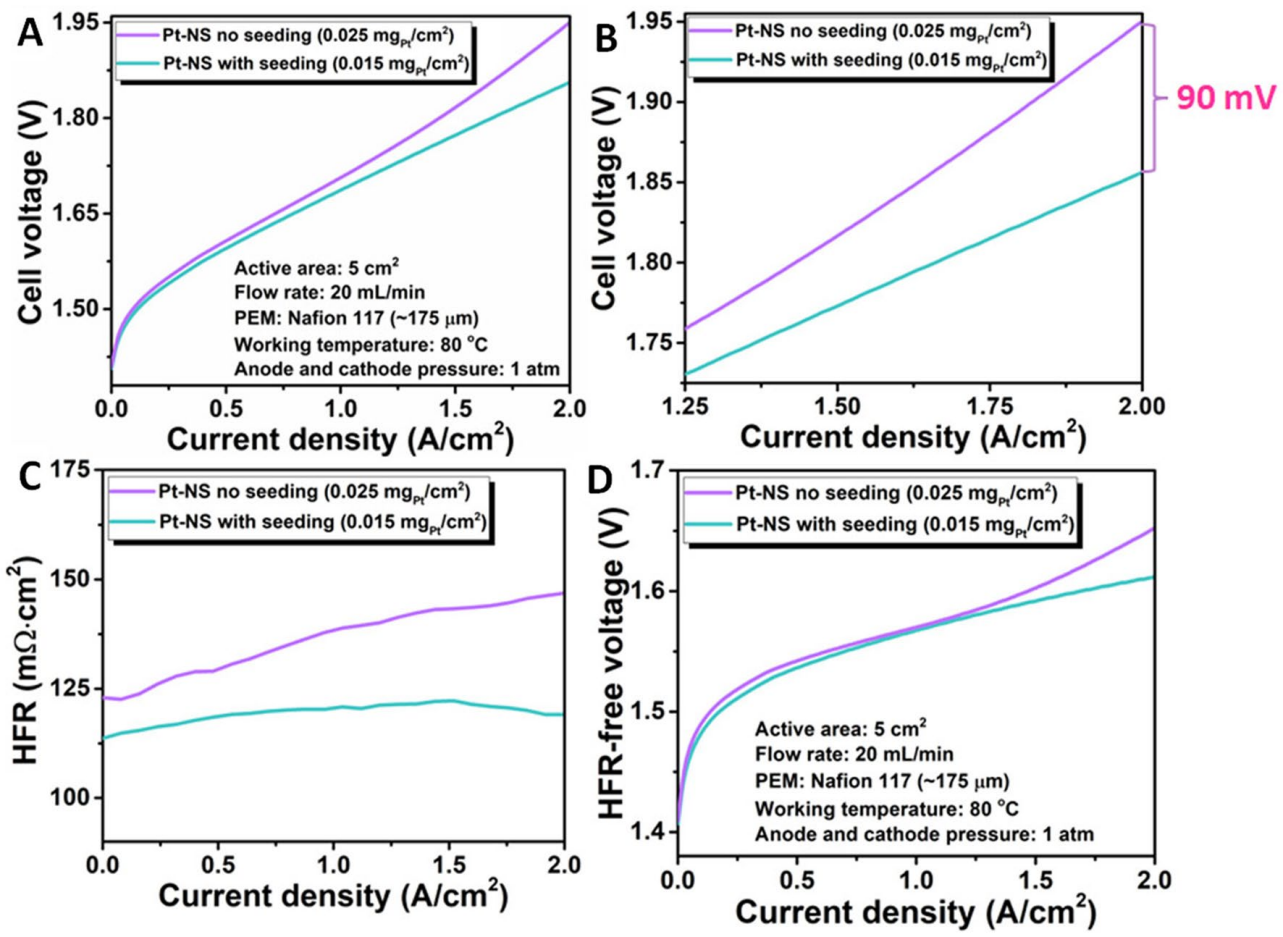
*In situ* cell performance comparison of Pt-NS with and without the seeding layer in a PEMEC at 80 °C. **A** Cell polarization curves of the Pt-NS electrodes with and without the seeding layer; **B** Enlarge cell polarization curves of the Pt-NS electrodes with and without the seeding layer within the current density range of 1.25 to 2 A cm^−2^; **C** The related high-frequency resistance (HFR) plots; **D** The HFR-free cell polarization curves

To compare the cell performances of electrodes with and without the seeding layer, the identical anode-only Nafion 117 membranes were used for practical PEMEC tests at 80 °C. The test results were collected and presented in Fig. 5. Impressively, with or without the seeding layer, the Pt-NS electrodes show remarkably different performances. As illustrated in Fig. 5A, with the same catalyst loading of 0.025 A cm^−2^, the seed-assisted electrode delivers a low cell voltage of 1.86 V at 2 A cm^−2^, which is 90 mV lower than that of the seed-free electrode (1.95 V) (Fig. 5B). In addition, when the current density is higher than 1.25 A cm^−2^, a curve-up phenomenon is observed in the polarization curve for the seed-free electrode, suggesting insufficient active sites for the high-current density cell operation. Moreover, as shown in Fig. 5C, an obviously increased HFR plot is also observed for the seed-free electrode within the test current density range of 0 to 2 A cm^−2^, increasing from 123 to 147 mΩ cm^2^. While almost stable HFR plot is presented for the seed-assisted electrode. For the HFR-free polarization curves, an obvious curve-up phenomenon is also observed for the seed-free electrode. Meanwhile, a higher HFR-free voltage of 1.65 V is presented, which is 40 mV higher than the seed-assisted electrode (Fig. 5D). Overall, the inferior performance and increased HFR plot of the seed-free electrode are mainly due to the poor Pt surface coverage, as shown in Fig. 2A, which cannot provide sufficient active sites for the electrochemical reaction, especially at the high current density regions.

**Fig. 6:**
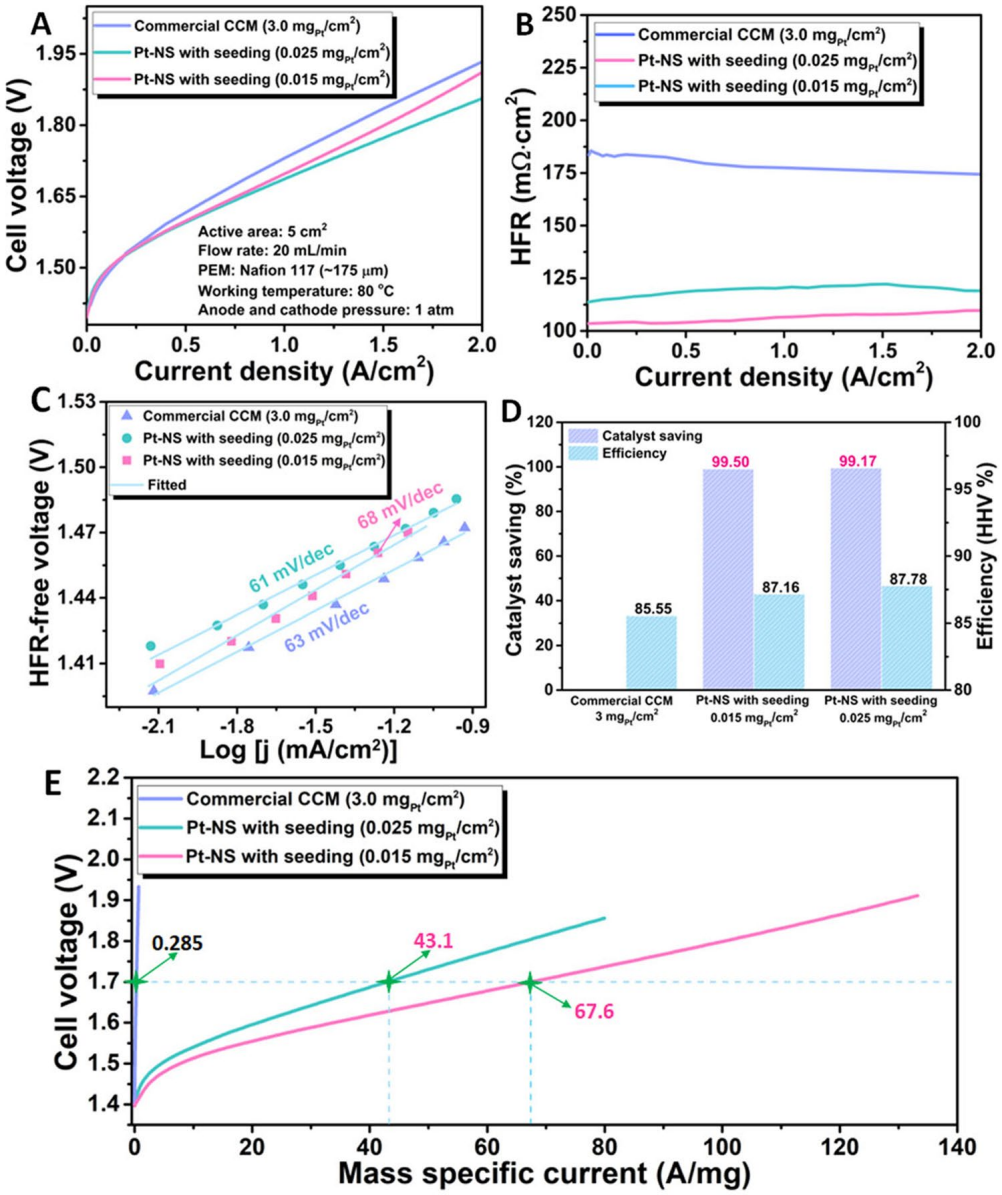
*In situ* cell performance comparison of the Pt-NS electrodes with different loadings (with the seeding layer) and the commercial CCM in a PEMEC at 80 °C. **A** Cell polarization curves of the Pt-NS electrodes and the commercial CCM; **B** The related HFR plots; **C** The corresponding Tafel plots; **D** Catalyst saving (Baseline: the CCM) and efficiency comparison at 1 A cm^−2^ of the Pt-NS electrodes and the commercial CCM; **E** The related Pt mass-normalized cell polarization curves

Based on the above results, the seeding layer plays a crucial role in the catalyst surface coverage and also cell performance. Hence, with the seeding layer, a lower Pt loading of 0.015 mg_Pt_ cm^−2^ is also prepared for the cell tests. Meanwhile, a commercial CCM with a Pt loading of 3.0 mg_Pt_ cm^−2^ was also evaluated as the baseline under identical operating conditions. Notably, with less than 0.9% catalyst loading could achieve a similar onset voltage of 1.40 V to the commercial CCM baseline (1.39 V). This could be because abundant active sites are exposed for the Pt-NS electrode, which is ascribed to the fine, vertically aligned Pt nanosheets with an ultrathin thickness of only 4 nm and small nanosheet sizes. Moreover, the Pt-NS electrodes show lower cell voltages of 1.86 V (0.025 mg_Pt_ cm^−2^) and 1.91 V (0.015 mg_Pt_ cm^−2^) at 2 A cm^−2^, respectively, which are superior to that of the commercial CCM (1.93 V) (Fig. 6A). As shown in Fig. 6B, the ionomer-free Pt-NS electrodes show low average HFR values of 117 mΩ cm^2^ for the 0.025-based electrode and 107 mΩ cm^2^ for the 0.015-based electrode, respectively, which are much lower than that of the commercial CCM (180 mΩ cm^2^). The low HFR values indicate the good conductivity of the Pt-NS electrodes, which could improve catalyst utilization, while the commercial CCM with a thick ionomer-involved catalyst layer shows high HFR values and limited conductivity, in which most of the catalysts are underutilized. To compare their activation loss differences, HFR-free polarization curves of Pt-NS electrodes and the baseline CCM are derived. As shown in Fig. S4, the HFR-free cell voltages of Pt-NS electrodes with 0.015 and 0.025 mg_Pt_ cm^−2^ and the baseline CCM are 1.69, 1.61, and 1.58 V, respectively, at 2 A cm^−2^. The activation loss of the 0.025-based electrode is slightly worse than the baseline CCM but better than the 0.015-based electrode. Such activation loss differences among the three electrodes are mainly due to the catalyst loading difference. Based on the HFR-free cell voltages, Tafel plots of different whole cells with different electrodes are derived. Since the anode setups for all cells are the same, the HER kinetics for the three cells are considered identical under the same HFR-free voltage; hence, the Tafel slope difference in the three cells should be only ascribed to the cathodes. As shown in Fig. 6C, similar Tafel slope values (0.025-based electrode: 61 mV dec^−1^; 0.015-based electrode: 68 mV dec^−1^; commercial CCM: 63 mV dec^−1^) are observed for all cells, indicating similar intrinsic catalyst activity of all electrodes in cell tests. Moreover, the similar Tafel slopes indicate that compared to the commercial CCM with an ionomer-mixed catalyst layer, there is no ion-conductivity issue for the Pt-NS electrodes with ionomer-free catalyst layer fabrication.

The cell efficiency and catalyst savings are compared as well in Fig. 6D. At the same current density of 1 A cm^−2^, both 0.025-based and 0.015-based Pt-NS electrodes display higher cell efficiencies of 87.16% and 87.78% than the baseline CCM (85.55%), achieving more than 99% catalyst savings over the CCM. To further evaluate the Pt catalyst utilization of different electrodes, mass-specific current, namely current density normalized by the Pt mass, was introduced. As shown in Fig. 6E, with the identical test conditions, the mass-specific currents of the Pt-NS electrodes with different loadings can reach up to about 80 A mg^−1^ (0.025 mg_Pt_ cm^−2^) and 133 A mg^−1^ (0.015 mg_Pt_ cm^−2^), which are much higher than that of the commercial CCM (0.666 A mg^−1^). The big differences demonstrate that compared to the commercial CCM, the ionomer-free Pt-NS electrode with ultrathin, fine, vertically aligned Pt nanosheets exposes abundant active reaction sites for the electrochemical reaction and achieves high catalyst utilization. At a specific cell voltage of 1.7 V, the 0.025-based and 0.015-based Pt-NS electrodes show high mass-specific currents of 43.1 and 67.6 A mg^−1^, respectively, impressively achieving about 151-fold and 237-fold higher than the commercial CCM (0.285 A mg^−1^). With the high catalyst utilization, the Pt loading can be significantly reduced with comparable performance, cutting down the PEMEC cost.

**Fig. 7:**
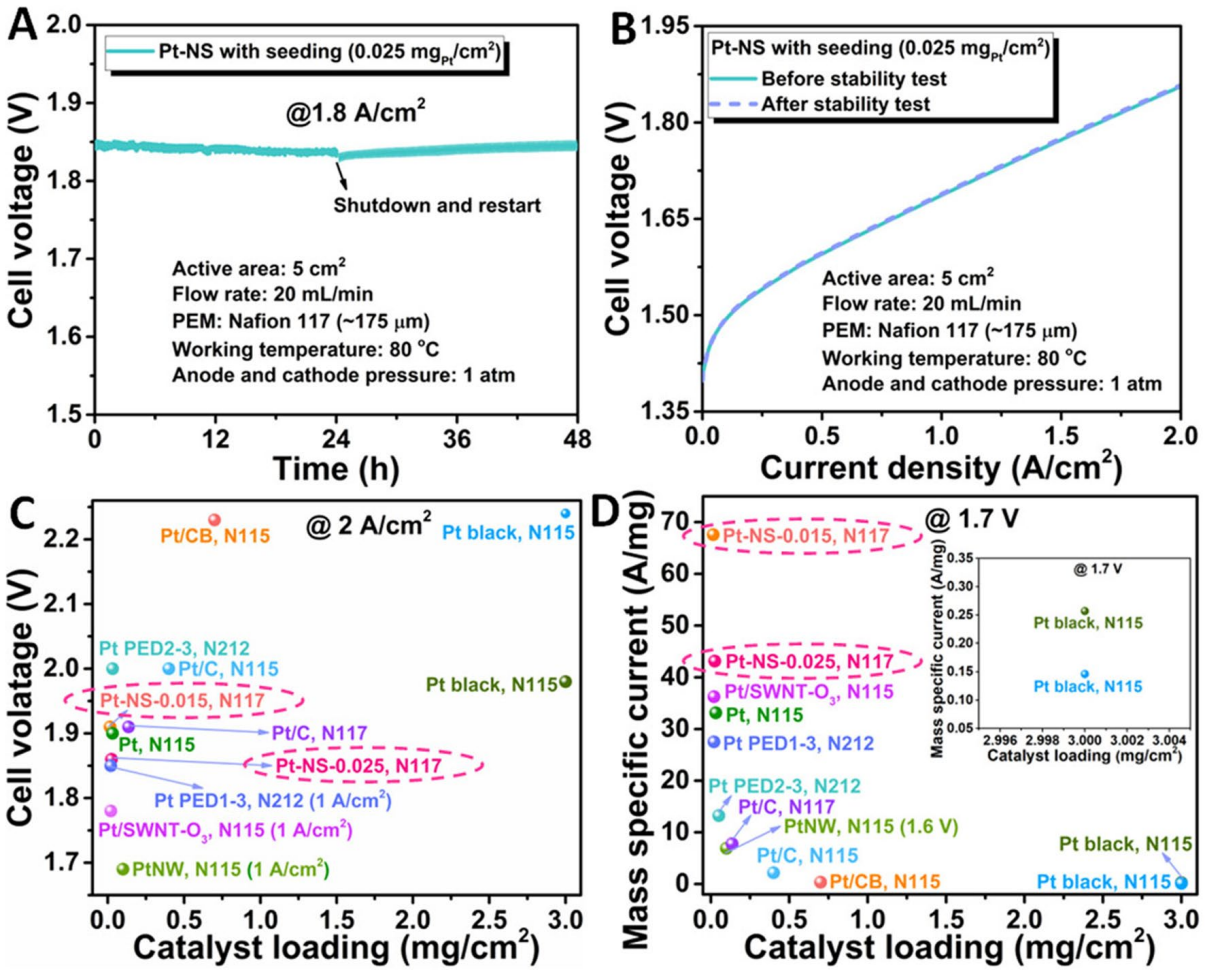
**A** Cell stability test at 1.8 A cm^−2^; **B** Cell performance comparison before and after the stability test; **C** Cell voltage comparison at 2 A cm^−2^ with previously reported cathode catalysts: Pt-NS-0.025, N117; Pt-NS-0.015, N117; Pt/C, N115 [14]; Pt/C, N115 [14]; Pt, N115 [20]; PtNW, N115 [24]; Pt PED1-3, N212 [25]; Pt PED2-3, N212 [25]; Pt/SWNT-O_3_, N115 [54]; Pt/C (0.4), N115 [55]; Pt/C (0.136), N117 [56]; Pt/CB, N115 [57]; **D** The corresponding mass-specific current comparison at 1.7 V; inset: enlarged area (the lower right) of the mass-specific current comparison at 1.7 V

In addition, the stability of the 0.025-based Pt-NS electrode was evaluated at a high current density of 1.8 A cm^−2^ for 48 h. As shown in Fig. 7A, the cell voltage of about 1.85 V is well maintained after the 48-h stability test. In addition, the polarization curves before and after the stability test overlap with each other in the current density range of 0 ~ 2 A cm^−2^, further demonstrating the excellent stability of the Pt-NS electrode in a practical PEMEC (Fig. 7B). It is noted that the performance of the Pt-NS electrode becomes slightly better during the initial 24-h stability test at 1.8 A cm^−2^, which is probably due to the gradual stabilization of the PEMEC at a high current density. A similar phenomenon is also presented in other studies [49–51]. However, after shutdown and restart, the performance gradually decreases in the second 24-h stability test. This opposite trend is probably attributed to the potential shielding effect of the generated hydrogen bubbles at the reaction interface between the Pt-NS electrode and solid electrolyte PEM, causing the slight decrease of reaction sites during the HER. After the 48-h stability test, as shown in Fig. 7B, the overlapping polarization curves before and after the stability test suggest that the observed slight performance loss in the second 24-h stability test is reversible. This phenomenon has been reported in previous studies [52, 53]. For example, Honsho et al. [52] reported that the reversible performance loss is most likely related to gas stagnation within the pores of the catalyst layers. Batalla et al. [53] reported that reversible voltage increase can be recovered when the cell current is interrupted. In addition, in the long-term perspective, coupling PEM water electrolysis with sustainable and renewable energy sources is highly appealing due to its high efficiency, close-to-zero emissions, and widespread applications. However, some renewable energy sources such as solar and wind power can be intermittent during practical applications. Thus, the main purpose of the shutdown and restart of the Pt-NS electrode integrated PEM electrolyzer cell during the stability test is to evaluate its response to an intermittent load. To further demonstrate the remarkable performance of the ultrathin Pt nanosheet-integrated thin electrodes, a cell performance comparison with the previously reported cathode catalysts for PEMECs was performed. As shown from Fig. 7C, even with a thicker Nafion 117 membrane (175 µm), the Pt-NS electrodes with ultralow Pt loadings in our work exhibit superior cell performance, achieving low cell voltages of 1.86 V for the 0.025-based and 1.91 V for the 0.015-based at 2 A cm^−2^. Additionally, significantly boosted catalyst utilization is also demonstrated, as evidenced by much higher mass-specific currents, of 43.13 A mg^−1^ for the 0.025-based and 67.60 A mg^−1^ for the 0.015-based at 1.7 V, than most of the recently reported cathode catalysts for the PEMEC (Fig. 7D) [14, 20, 24, 25, 54–57].

The details about different electrode fabrications are listed and compared in Table 1. Compared to complex fabrication methods involving multiple steps and expensive equipment in most recently reported studies, in this work, a facile electrochemical growth process with high efficiency (≤ 25 s) was adopted to fabricate the electrodes at room temperature with ultralow loadings (≤ 0.025 mg_Pt_ cm^−2^) and ultrathin vertically aligned Pt nanosheets, without the use of any surfactants, templates, pH adjusters, and elaborate equipment. This electrochemical growth process offers an ionomer-free catalyst layer with good conductivity and is much more efficient and facile than most studies. For example, Rajala et al*.* [54] prepared platinum nanowires on ozonized single-walled carbon nanotubes (SWNT-O_3_) for the PEMEC, employing multiple fabrication procedures with high cost. Specifically, expensive SWNTs were functionalized via an ozone generator as the supporters, and then, Pt catalysts were modified on the SWNT-O_3_ to obtain Pt/SWNT-O_3_ at a high temperature of 300 °C under a 5% H_2_/Ar atmosphere. Afterward, the obtained Pt/SWNT-O_3_ catalysts were prepared into catalyst ink and then sprayed onto a carbon paper, which involved ionomers in the catalyst layer offering limited conductivity. Moreover, a high annealing temperature of 300 °C and expensive equipment such as a spray coater was needed for the electrode fabrication. Even with a one-step fabrication method such as pulse electrodeposition or sputtering, the catalyst morphology and performance of the prepared electrode are limited. For example, Park et al*.* [25] adopted a pulse electrodeposition method to prepare the electrode, which exhibited poor catalyst surface coverage and resulted in an inferior performance of ≥ 1.85 V at 1 A cm^−2^ even with a much thinner membrane of Nafion 212 (~ 50 µm). Overall, the highly efficient and sustainable electrode fabrication with high performance and low cost demonstrated in our work, which enables easy electrode fabrication scale-up, could efficiently accelerate PEMEC large-scale applications.

**Table 1:** Details of different electrode fabrications

Refs./cathode (mg_Pt_ cm^−2^)	Cathode fabrication	Catalyst structure/catalyst surface coating	Fabrication steps/time	Elaborate or additional equipment	With or without ionomer
This work/Pt-NS (0.025)	Electrochemical growth	Ultrathin nanosheets/uniform coating	One-step/ < 25 s	No	Ionomer-free
This work/Pt-NS (0.015)	Electrochemical growth	Ultrathin nanosheets/uniform coating	One-step/ < 25 s	No	Ionomer-free
[14]/Pt black (3)	Commercial Pt black + spray	Nanoparticles/–	Multiple complex steps	Spray coater	With ionomer
[14]/Pt black (3)	Commercial Pt black + spray + decal	Nanoparticles/–	Multiple complex steps	Spray coater, hot press	With ionomer
[20]/Pt (0.032)	Sputtering	Nanoparticles/uniform coating	One-step/45 s	Sputter coater	Ionomer-free
[24]/PtNW (0.1)	Wet chemical reduction	Nanowires/uniform coating	One-step/16 h	No	Ionomer-free
[25]/Pt PED1-3 (0.020)	Pulse electrodeposition	Flower-like structures/nonuniform coating	One-step/300 s	No	Ionomer-free
[25]/Pt PES2-3 (0.051)	Pulse electrodeposition	Polyhedral particles/nonuniform coating	One-step/300 s	No	Ionomer-free
[54]/Pt/SWNT-O3 (0.02)	Ozonized SWNT preparation + annealing in H_2_/N_2_ at 300 ℃ + spray	Nanowires/–	Multiple complex steps	Ozone generator, tube furnace, spray coater	With ionomer
[55]/Pt/C (0.4)	Commercial Pt/C + spray	Nanoparticles/–	Multiple complex steps	Spray coater	With ionomer
[56]/Pt/C (0.136)	Commercial Pt/C + spray	Nanoparticles/–	Multiple complex steps	Spray coater	With ionomer
[57]/Pt/CB (0.7)	Modified Adams fusion method (annealing at 500 °C) + spray	Nanoparticles/–	Multiple complex steps	Muffle furnace, spray coater	With ionomer

### Post-analysis of the Pt-NS Electrode after the Cell Test

**Fig. 8:**
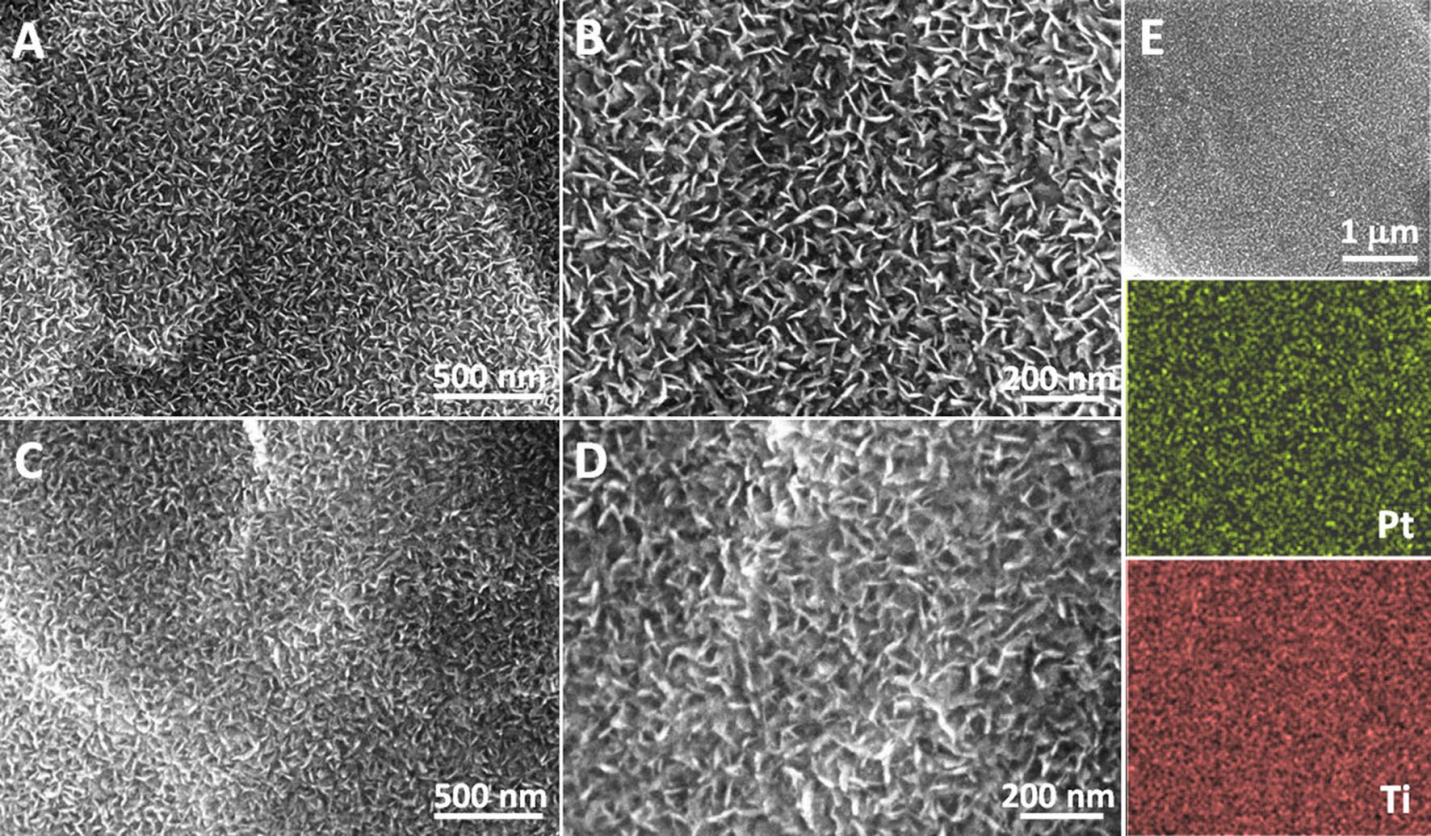
Morphology comparison before and after the *in situ* cell stability test. SEM images of the **A**,** B** fresh and **C**, **D** tested Pt-NS electrode; **E** SEM–EDS mapping images of the tested Pt-NS electrode

Post-test characterizations of the Pt-NS electrode (0.025 mg_Pt_ cm^−2^), including SEM and SEM–EDS mapping and analysis, were performed to check Pt nanosheet morphology and composition changes. As seen from the SEM images in Fig. 8A–D, the fine nanosheet morphologies are well-maintained after the 48-h stability test at the high current density of 1.8 A cm^−2^. In addition, the SEM–EDS mapping results show that uniform Pt surface coverage is still observed on the Ti substrate (Fig. 8E). Meanwhile, based on the EDS result, the test Pt-NS electrode exhibits a similar Pt content (4.31%) with the fresh one (Fig. S5), indicating that there is no significant catalyst loss from the substrate after the cell test. Overall, the above results demonstrate the good stability of the prepared Pt-NS electrode in a practical PEMEC.

**Fig. 9:**
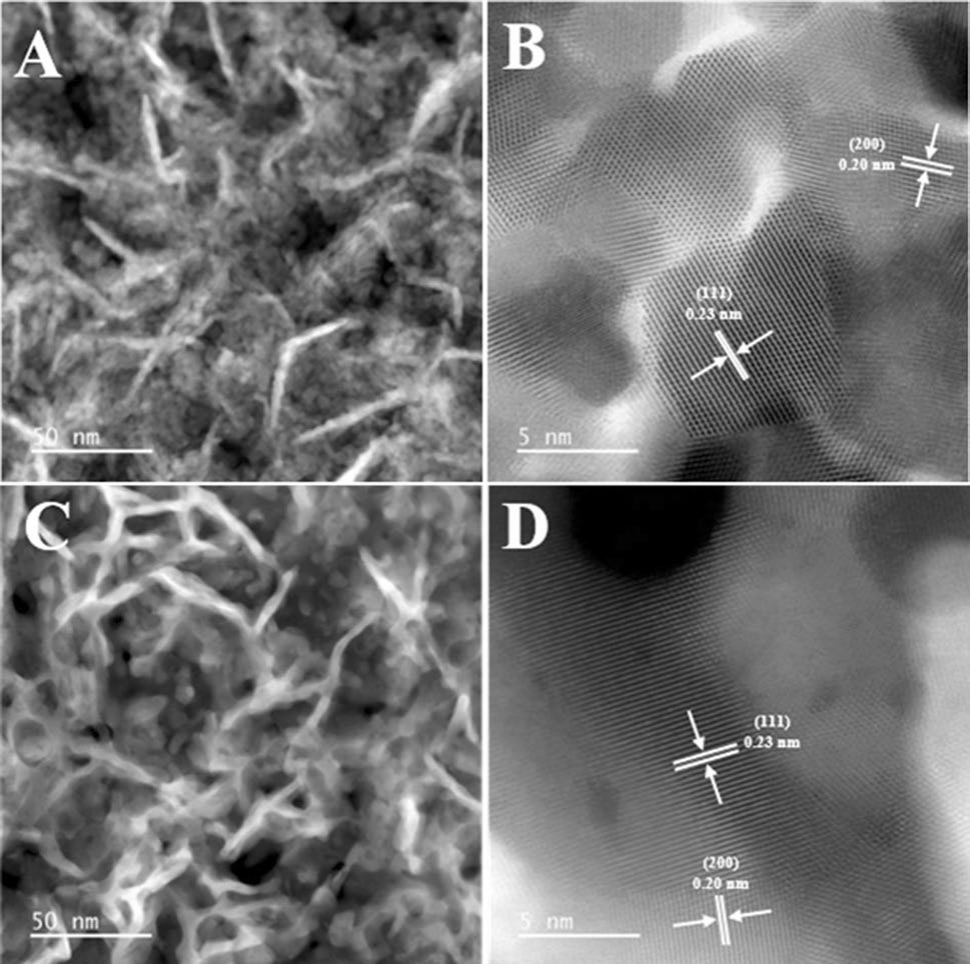
High-angle annular dark-field (HAADF) STEM images of **A**, **B** the fresh and **C**, **D** tested Pt-NS electrode

Meanwhile, to further investigate any potential structural and crystal changes of Pt nanosheets that may occur before and after the stability test, we prepared samples by scraping the Pt nanosheets from the electrode surface and characterized both fresh and tested samples using HAADF-STEM imaging. Figure 9A confirms the well-defined structure of the nanosheets of the fresh sample and indicates that the thickness of individual platinum nanosheets is approximately 4 nm. As shown in Fig. 9B, the lattice spacings of 0.23 and 0.20 nm are determined, corresponding to the crystal planes of Pt(111) and Pt(200), respectively. Furthermore, the HAADF-STEM images of the tested sample in Fig. 9C, D reveal that the nanosheet structure and crystal structure have been preserved even after the stability test at a high current density of 1.8 A cm^−2^. These findings are in good accordance with the results from Fig. 8, which offer compelling evidence for the good structural stability of the as-obtained Pt nanosheets.

## Conclusions

In summary, we developed vertically well-aligned Pt nanosheets (Pt-NSs) with bottom-up growth on thin Ti substrates, showing nanoscale catalyst layers (≤ 50 nm) and full surface coverage even at ultralow catalyst loadings via a facile electrochemical growth process at room temperature. The fabricated ultralow-loaded Pt-NSs exhibit ultrathin thicknesses of only about 4 nm, small nanosheet sizes, and vertically well-aligned nanosheet morphologies. Moreover, the entire electrode fabrication process is very rapid, environmentally friendly, and easily scalable and occurs without the use of any surfactants, templates, pH adjusters, and elaborate equipment. When coupled with an anode-only catalyst-coated Nafion 117 membrane, the Pt-NS electrode with an ultralow Pt loading of 0.015 mg_Pt_ cm^−2^ shows superior cell performance to the commercial CCM (mg_Pt_ cm^−2^), achieving 99.5% catalyst savings and more than 237-fold higher catalyst utilization. Overall, this study could not only guide uniform catalyst coating on substrates at ultralow loadings but also pave a new way for developing nanostructured electrodes with nanoscale catalyst layers for energy-saving PEMECs and other energy storage/conversion devices.

### Supplementary Information

Below is the link to the electronic supplementary material.

Supplementary file1 (PDF 803 KB)

Supplementary file2 (MP4 26321 KB)

Supplementary file3 (MP4 26369 KB)
