# Supplementary material for: Electrochemically Grown Ultrathin Platinum Nanosheet Electrodes with Ultralow Loadings for Energy-Saving and Industrial-Level Hydrogen Evolution
Source: Nanomicro Lett. 2023 Jun 3;15:144. doi: 10.1007/s40820-023-01117-2 (PMC10239421; doi:10.1007/s40820-023-01117-2)
Supplement: Supplementary file 1 — Supplementary file1 (PDF 803 KB) [file 40820_2023_1117_MOESM1_ESM.pdf]

Supporting Information for

Electrochemically Grown Ultrathin Platinum Nanosheet Electrodes with Ultralow Loadings for Energy-Saving and Industrial-Level Hydrogen Evolution

Lei Ding[1, #], Zhiqiang Xie[1, #], Shule Yu[1, #], Weitian Wang[1], Alexander Y. Terekhov[2], Brian K. Canfield[2], Christopher B. Capuano[3], Alex Keane[3], Kathy Ayers[3], David A. Cullen[4], and Feng-Yuan Zhang[1, *]

[1] Nanodynamics and High-Efficiency Lab for Propulsion and Power, Department of Mechanical, Aerospace & Biomedical Engineering, UT Space Institute (University of Tennessee-Knoxville), Tullahoma, TN 37388, USA

[2] Center for Laser Applications, UT Space Institute (University of Tennessee-Knoxville), Tullahoma, TN 37388, USA

[3] Nel Hydrogen, Wallingford, Connecticut 06492, USA

[4] Center for Nanophase Materials Sciences, Oak Ridge National Laboratory, Oak Ridge, TN 37831, USA

[#]Lei Ding, Zhiqiang Xie, and Shule Yu contributed equally to this work.

*Corresponding author. E-mail: fzhang@utk.edu (F.-Y. Zhang)

Supplementary Figures and Table

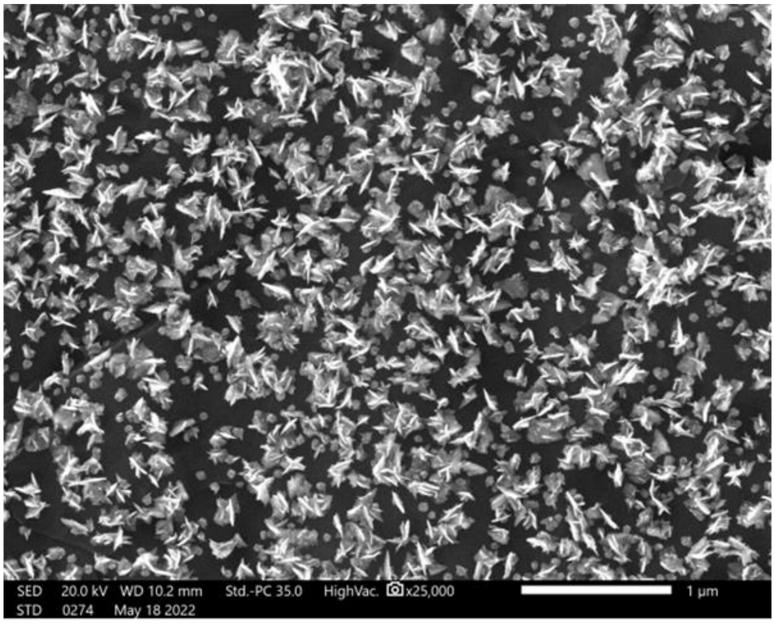

Fig. S1 SEM image of Pt-NSs without the seeding layer: 0.07 mg_{Pt}/cm^2 Pt-NSs

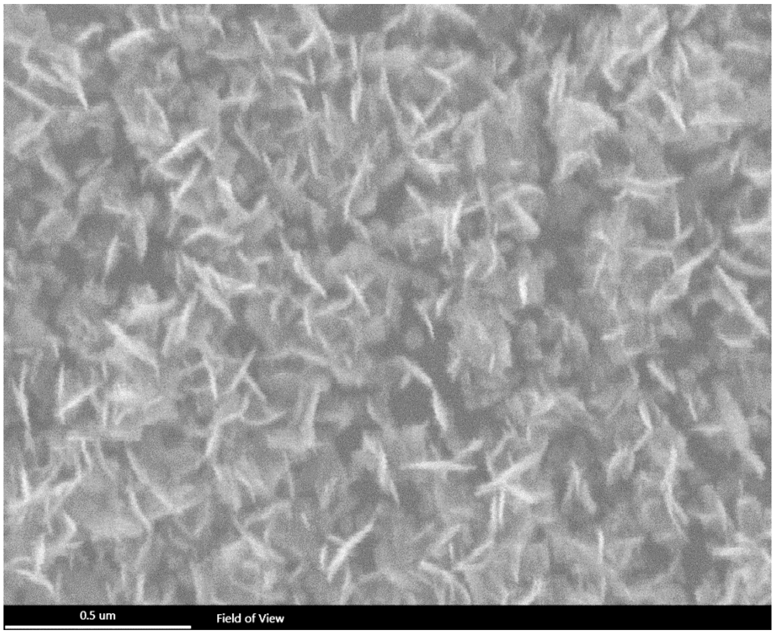

Fig. S2 SEM image of Pt-NSs without the seeding layer: 0.14 mg_{Pt}/cm^2 Pt-NSs

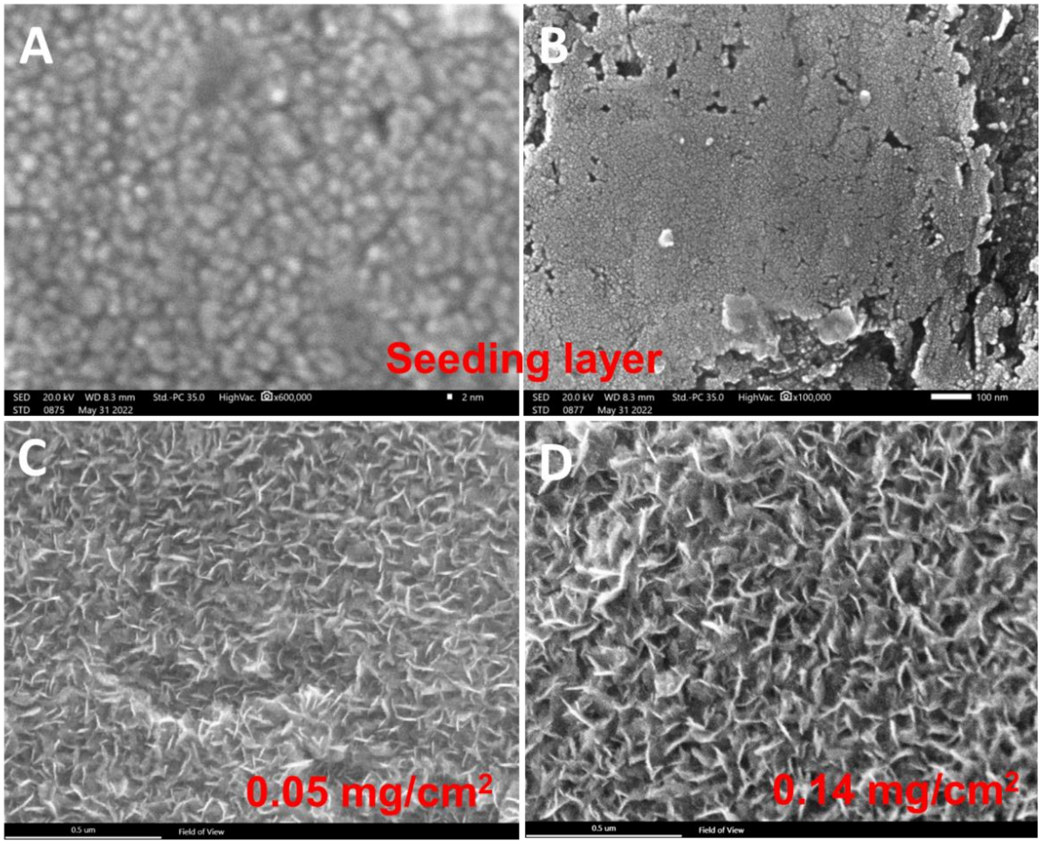

Fig. S3 SEM images of Pt nanoparticle seeding layers (**A** and **B**); SEM images of Pt-NSs with the seeding layer, (**C**) 0.050 mg_{Pt}/cm^2 Pt-NSs, (**D**) 0.140 mg_{Pt}/cm^2 Pt-NSs

Table S1 EIS fitting parameters derived from the Nyquist plots in **Fig. 4C**

Electrode	R_{Ω} [mΩ·cm^2]	R_{hf} [mΩ·cm^2]	Q_{hf} [mF·s^{n-1}/cm^2]	n_{hf}	R_{lf} [mΩ·cm^2]	Q_{lf} [mF·s^{n-1}/cm^2]	n_{lf}	$C_{dl,hf}$ [mF/cm^2]	$C_{dl,lf}$ [mF/cm^2]	Error (%)
**Pt-NS**	2.06	1.75	85.46	0.600	0.416	5.39	0.766	23.5	0.833	0.012
**Pt-NP**	2.14	3.26	63.7	0.345	1.87	138	0.819	3.26	115	0.023

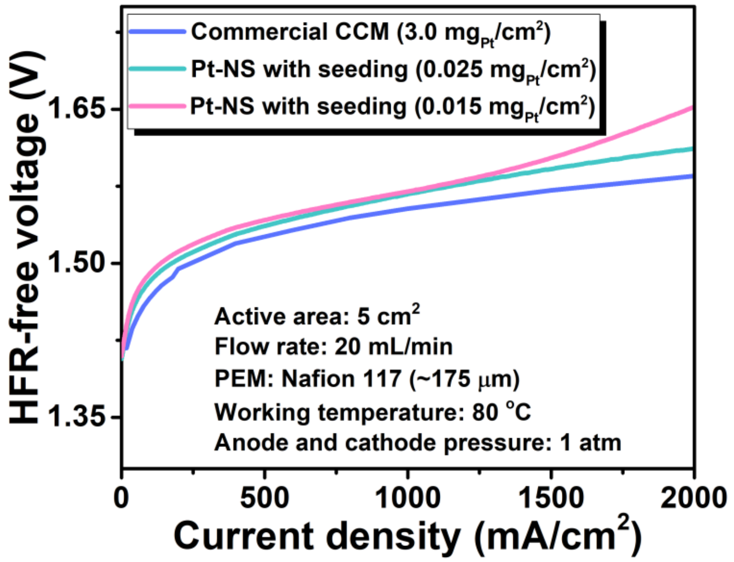

Fig. S4 HFR-free cell polarization curves of commercial CCM and Pt-NS with seeding layer: 0.025 mg$_{Pt}$/cm^2 and 0.015 mg$_{Pt}$/cm^2

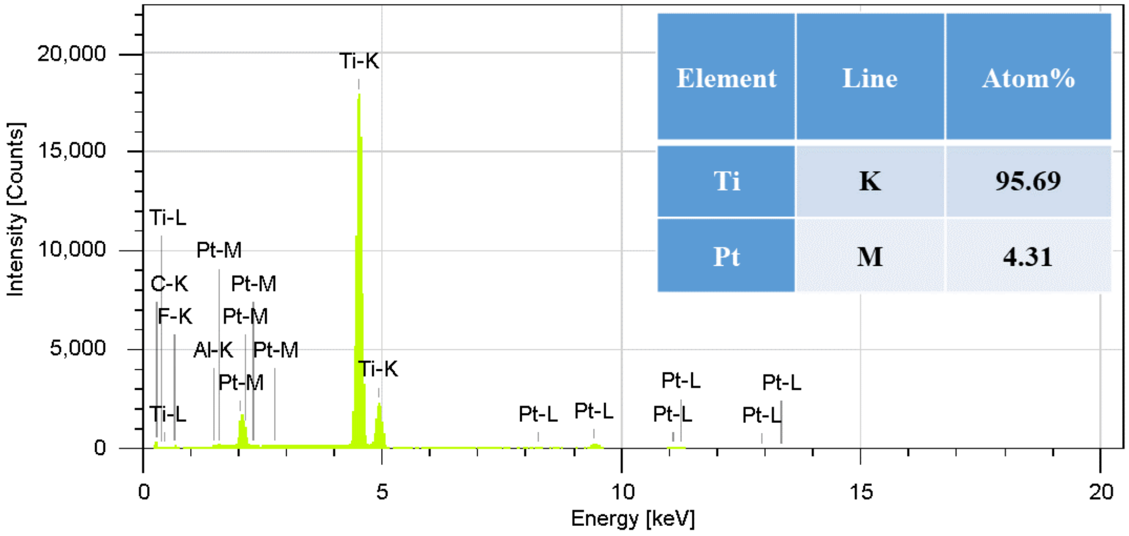

Fig. S5 EDS analysis of the tested Pt-NS CCLGDL (0.025 mg$_{Pt}$/cm^2)
